# Extravasation d'un produit de chimiothérapie

**DOI:** 10.11604/pamj.2016.24.55.8930

**Published:** 2016-05-13

**Authors:** Sami Aziz Brahmi, Fatima Zahra Ziani

**Affiliations:** 1 Service d'Oncologie Médicale, Centre Hospitalier Mohammed VI, Oujda, Maroc; 2 Service d'Oncologie Médicale, Centre Hospitalier Hassan II, Fès, Maroc

**Keywords:** Extravasation, chimiothérapie, vesicant, Extravasation, chemotherapy, vesicant

## Image en médecine

L'extravasation en oncologie est définie comme une fuite accidentelle au cours de la perfusion d'unproduit de chimiothérapie, cette fuite peut se produire sur la peau, les tissus annexes et elle est considérée comme une urgence thérapeutique. Nous présentons l'image d'une extravasation d'un produit de chimiothérapie (la vinorelbine) survenue chez un patient de 50 ans suivie pour cancer du poumon métastatique , le patient a été perfusé au niveau de la face dorsale de la main, il s'est présenté 3 jours après la cure avec œdème et phlyctènes de la face dorsale de la main, douleur et rougeur accentués au niveau du point d'injection du produit. Le patient a été mis sous corticoïdes topiques et antalgiques avec une bonne évolution. La vinorelbine appartient à la famille des vinca-alcaloides, c'est un produit vésicant avec un risque de nécrose cutanée. Le praticien doit être avertit du risque et le patient doit être informé et éduqué. Une prise en charge rapide au moment de l'accident est nécessaire suivant un protocole établi par l’équipe soignante.

**Figure 1:**
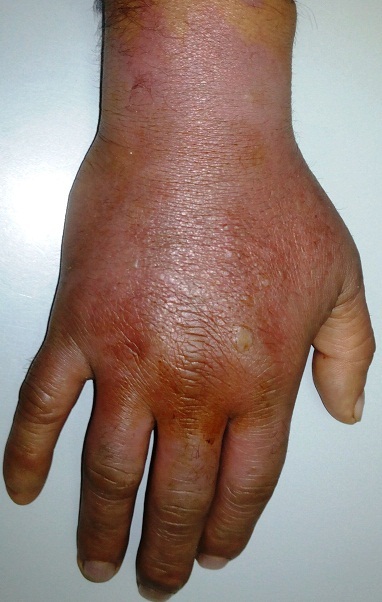
Extravasation de la face dorsale de la main

